# Specificity, Co-Occurrence, and Growth: Math and Reading Skill Development in Children With Learning Disabilities

**DOI:** 10.1177/00222194241312189

**Published:** 2025-02-09

**Authors:** Katherine Helene Connors, Emily L. Guertin, Melissa Nichol, Joan M. Bosson-Heenan, Jeffrey R. Gruen, Jan C. Frijters

**Affiliations:** 1 Brock University, St. Catharines, ON, Canada; 2 Yale School of Medicine, New Haven, CT, USA

**Keywords:** learning disability, comorbidity, co-occurring disability, math disability, reading disability, growth curve analysis, linear mixed effects models, longitudinal design

## Abstract

Learning disabilities are challenging to characterize because they evolve throughout development, frequently co-occur, and have varying domain specificity. Addressing these challenges, we analyzed longitudinal patterns of growth, co-occurrence, and specificity manifesting in the math and reading skills of children with and without learning disabilities. With a sample of 498 Grade 1 U.S. children followed for 5 years, we used linear mixed-effects models to explore group-level differences among children with math disability (MD), reading disability (RD), co-occurring disability, and no disability. Findings revealed: Math and reading trajectories of children with learning disabilities parallel those of children without disabilities. Skill growth slows over time, regardless of skill level, suggesting disability-related impairments will not resolve without intervention. Impairment levels and growth trajectories of children with co-occurring disabilities match the within-domain patterns of children with isolated disabilities, supporting a longitudinally maintained additive model of co-occurrence. MD and RD show varying specificity. MD impairments are domain-specific and become more pronounced over time. RD impairments impact both domains early, become more domain-specific over time, but maintain curriculum-contingent math deficits. Findings suggest early math intervention should balance linguistic and conceptual support, as the source of a child’s math difficulties may not be clear until well into elementary school.

Learning disabilities have long been of interest to a wide range of stakeholders: parents, educators, clinicians, researchers, pediatricians, and most importantly, the children, adolescents, and adults who experience them. The field of learning disabilities marries the complexities of psychology, biology, and sociology with the nuances of human performance and developmental change. Several decades of robust work in this field has revealed three sources of complexity that make characterizing learning disabilities a unique challenge: (a) their symptomatology changes across development, (b) they frequently co-occur, manifesting simultaneously in multiple academic domains, and (c) the skills they impact are domain-specific in some cases but domain-general in others. This paper seeks to explore these complexities by focusing on how several key math and reading skill impairments associated with learning disabilities manifest over time.

## Longitudinal Patterns of Math and Reading Skill Development

Longitudinal studies conducted with varying age ranges and populations have found that math and reading share many developmental similarities. Growth rates on both domains tend to decelerate over development, with most rapid growth happening before Grade 3 (Cameron et al., 2015; Little et al., 2021; Peralta et al., 2023). Math skill can be used to predict future reading progress (Duncan et al., 2007; Lerkkanen et al., 2005), and reading ability has been shown to support math growth (Lin & Powell, 2022; Shin et al., 2013; Singer et al., 2019). There is ample evidence in each of these studies that math and reading growth is interrelated.

Despite strong interrelation, math and reading trajectories do have differences. A recent study by Peralta et al. (2023) found that reading and math growth trajectories from kindergarten to middle school were positively correlated, but the strength of that relationship decreased over time. This suggests that there is more domain specificity at later stages of development than there is at earlier ones. Little et al. (2021) found that individual differentiation in reading ability was strong in early elementary (Grades K–2) but non-significant in late elementary (Grades 3–5). They found the inverse with math, where individual differences did not emerge until late elementary. One implication of the distinct timelines of individual differentiation in math and reading is that disability-related impairments in each domain may manifest in different timelines.

Collectively, longitudinal studies of math and reading suggest that there are both shared cross-domain and unique domain-specific patterns of math and reading skill development. Our study is interested in exploring how these growth patterns manifest in the presence and absence of math and reading learning disabilities.

## Co-Occurrence of Math and Reading Disability

Shifting operational definitions make it difficult to ascertain prevalence and incidence rates for learning disabilities. A 2020 article by Grigorenko et al. summarizing the collective insight gained over 50 years in specific learning disabilities research reports prevalence estimates in the range of 4%–8% for math disability (MD), and 5%–17% for reading disability (RD). An estimated 30%–50% of children with reading difficulties also struggle with math (Grigorenko et al., 2020; Moll et al., 2014). While exact co-occurrence rates are elusive, it is clear that the incidence of co-occurring RD and MD is greater than that which would be expected when considering the incidence of each disability alone (Dirks et al., 2008).

Some models have suggested that co-occurring MD and RD is largely the result of shared underlying general impairments which include verbal comprehension difficulty, slow processing speed, and problems with working memory (Peterson et al., 2017; Willcutt et al., 2013). Dirks et al. (2008) found that children with co-occurring MD and RD exhibited more generalized achievement difficulty than those with single disabilities (i.e., disability manifesting primarily in one domain). Peterson et al. (2017) found that verbal comprehension difficulty was a common impairment for MD and RD. They also confirmed previous findings that slow processing speed served as a common impairment for MD, RD, and attention-deficit/hyperactivity disorder (ADHD).

There is strong empirical evidence that MD and RD are distinct but related disorders, and their co-occurrence can be characterized as the additive result of MD-related difficulties plus RD-related difficulties (Grant et al., 2020; Moll et al., 2016; Willcutt et al., 2013; Wilson et al., 2015; Wong & Ho, 2021). Most of these studies have explored co-occurring MD and RD with a focus on general cognitive processes that are implicated in both domains. Our study is interested in looking at how co-occurring disability manifests over time in the reading and math skills themselves, with attention paid to whether developmental patterns differ from isolated disability.

## Specificity in Math and Reading Learning Disabilities

Even when MD and RD occur in isolation, their specificity is not necessarily a given. The specificity of learning disabilities has been a topic of debate in the field almost since its inception (Compton et al., 2012; Fletcher et al., 2019). A longstanding question in the field has centered around the degree to which difficulties associated with learning disabilities are the result of specific or general impairment. Early models of learning disability favor specificity, proposing that a specific learning disability is the result of a single domain-specific underlying cognitive deficit. The most common such model in RD is the phonological theory, which attributes RD to impairment in speech-sound processing (Brady et al., 1983; Ramus et al., 2003; Snowling, 1981). The MD literature has presented diverse frameworks of single impairment, implicating poor number sense (Feigenson et al., 2004; Fuchs et al., 2010), fact-retrieval difficulty (Geary et al., 2012; Jordan et al., 2003), difficulty disregarding task-irrelevant information (Geary, 2011), difficulty processing or manipulating number sets (Geary et al., 2004; Koontz & Berch, 1996), and counting difficulty (Geary et al., 1992).

Increasingly, there is evidence that both domain-specific and general deficits are implicated in learning disabilities. Fuchs et al. (2010) found number sense to be the primary predictor of calculation difficulties but identified general factors, such as language, attention, and non-verbal problem-solving, as playing meaningful roles in difficulties with mathematical word problems. Landerl and Moll (2010) found that both domain-specific and generalized deficits were implicated in reading, math, and spelling disabilities. A longitudinal study by Pennington et al. (2012) found that performance patterns in a sample of participants with RD were best captured by a model that included multiple paths to disability, some tracing to highly specific impairments and others to a more general array of impairments.

The learning disabilities field has moved toward conceptualizing learning disabilities as probabilistic in nature, as opposed to being neatly and deterministically defined by universal core impairments (Astle & Fletcher-Watson, 2020; Lervåg, 2021; Moll 2022). Learning disabilities involve various combinations of underlying cognitive impairments that interact with various non-cognitive risk factors in diverse ways (Bishop & Snowling, 2004; Pennington, 2006; van Bergen et al., 2014). RD and MD tend to be especially heterogeneous, manifesting with so much variety in impairment profiles that it can be difficult to characterize meaningful subtypes (Huijsmans et al., 2020; Wagner et al., 2020). Somewhat paradoxically, learning disabilities can appear quite distinctive at the individual level and rather nebulous at the group level. The last few decades of research have answered the question of whether learning disabilities are *specific* or *general*—it seems they are both. But is the balance of specificity and generality similar for all learning disabilities? Our study seeks to explore this in the context of within- and cross-domain skill development in children with isolated MD and RD.

## The Present Study

In the present study, we utilized linear mixed-effect models to investigate patterns of math and reading sub-skill growth in longitudinal data from a demographically and academically diverse group of children. We framed analyses around three research questions, addressed by conducting orthogonal comparisons on groups with increasingly specific disability profiles.

1. Are the math and reading growth trajectories of children with learning disabilities similar to those of children without disabilities? To address this, we compared children with any evidence of math and/or reading disability to those without.
2. Are the growth trajectories of children with co-occurring MD and RD similar to those of children with single-domain disabilities? To address this, we compared children with co-occurring MD and RD to those with a single disability in either.
3. Do children with single MD and single RD show varying degrees of specificity in their developmental patterns of impairment? To address this, we compared children with single MD to those with single RD.

## Method

The present analyses utilized data from the New Haven Lexinome Project (NHLP). The NHLP is a large longitudinal study which partners Yale University and New Haven Public Schools in assessing reading and cognitive development via collection of genetic, neuroimaging, and psychometric data. The present analyses focused solely on psychometric and demographic data.

Psychometric data were collected from study participants over the course of 5 years by a team of trained psychometrists. Data were collected in two waves, with first and second cohorts entering the study in Fall of 2015 and Fall of 2016, respectively. Entry into the study was open to any first-graders enrolled in New Haven Public Schools. Study information was distributed via school newsletter and email to families of potential enrollees, and interested parents/guardians were invited to contact NHLP personnel for information and screening. All interested individuals were invited to enroll, unless they met any of the following exclusionary criteria: frequent school absences (>20 days) during the previous kindergarten year, and/or diagnosis of neurological disorder (e.g., autism spectrum disorder, fetal alcohol syndrome, brain injury, seizure disorder, etc.). Participants were not excluded for having other co-occurring developmental disorders (e.g., developmental language disorder, motor and coordination disorder) or attention disorders (i.e., ADHD).

Upon entry, parents or guardians of participating children completed a questionnaire to gather demographic data. Reading and math performance data were then collected annually (math) or semiannually (reading) for 5 years. The tenth and final reading measurement point for first-cohort participants and the final two data points for second-cohort participants were not gathered due to school shutdowns in response to COVID-19. In all, the data included in this study feature nine measurement points for reading and five measurement points for math.

### Participants

Analyses were conducted on a sample of 498 children, spread across 32 schools in a historically under-resourced urban area. The mean age at entry into the study (Fall of Grade 1) was 6.61, and the mean IQ as measured by the Wechsler Abbreviated Scale of Intelligence-Second Edition (WASI-II) Perceptual Reasoning Index (PRI) was 91. Demographically, the sample is as follows: sex—54% female (*n* = 267), 46% male (*n* = 231); socioeconomic status (SES)—89% qualify for government assistance (*n* = 444); language exposure—50% have evidence of daily exposure to/use of a non-English language (*n* = 248); race and ethnicity as reported by participant’s parent/guardian (able to select as many categories as apply)—1.2% Asian (*n* = 6), 0% American Indian or Alaska Native (*n* = 0), 31% Black or African American (*n* = 153), 49% Hispanic (*n* = 242), 25% White or Caucasian (*n* = 125), 1.2% Native Hawaiian or Other Pacific Islander (*n* = 6), 18% did not report race and ethnicity information (*n* = 88). In relation to publicly available demographic information about the school district, the sample’s racial and ethnic makeup is as expected, but it appears to have comparatively high representation of low-SES participants.

Prior to conducting our main analyses, participants were classified into four orthogonal disability groups: (a) Single Reading Disability (RD-only)—participants exhibiting persistent difficulty in only the reading domain (15%, *n* = 74); (b) Single Math Disability (MD-only)—participants exhibiting persistent difficulty in only the math domain (5%, *n* = 27); (c) Co-occurring Math and Reading Disability (MD & RD)—participants exhibiting persistent difficulty in both math and reading domains (26%, *n* = 127); and (d) No Disability—participants who did not exhibit persistent difficulty in either domain (54%, *n* = 270).

Our sample includes a much larger percentage of children with learning disabilities than would be found with random sampling. RD was an advertised focus of the NHLP project, which led to the expected overrepresentation of children with RDs. Due to co-occurrence, this expanded to overrepresentation of learning disabilities more generally. The high percentage of learning disability may also be related to the socioeconomic makeup of the sample, as low SES is known to be associated with higher likelihood of learning disability identification (Shifrer et al., 2011).

Table 1 reports descriptives for each disability group and the sample as a whole. In our growth models, IQ, SES, language exposure, sex, ethnicity, and race were included as covariates to control for their contribution to group differences. However, we also ran analyses on each characteristic to identify where and how the four disability groups differed on these features. For the continuous variable IQ, a one-way analysis of variance (ANOVA) showed no significant differences between any of the groups, *F*(3, 494) = 0.746, *p* = .525, suggesting comparable cognitive abilities across disability groups. A chi-square test on language exposure showed marginally significant differences across groups (χ²(3) = 7.745, *p* = .052), the source of which was a significant difference in language exposure between the MD-Only and RD-Only groups, with the MD-Only group having the highest proportion of children with frequent exposure to a language other than English. Significant group differences were found on SES (χ²(3) = 12.449, *p* = .006), the source of which was a larger proportion of low-SES students in the Co-occurring group than in the No Disability group. There was no significant association between sex and disability group (χ²(3) = 1.787, *p* = .618), suggesting that sex was not a distinguishing factor in the prevalence of MD and RD in our sample. Analysis of ethnic and racial distribution across the four disability groups showed no significant differences in self-reported ethnicity (χ²(3) = 4.153, *p* = .245), African American or Black racial identity (χ²(3) = 6.945, *p* = .074), nor White or Caucasian racial identity (χ²(3) = 6.707, *p* = .082). We were unable to do group analyses with participants identifying exclusively as Asian, American Indian or Alaska Native, or Native Hawaiian or Other Pacific Islander due to low representation in our sample.

**Table 1.** Full Sample and Disability Group Characteristics.

Characteristic	Full sample (*n* = 498)	No disability (*n* = 270)	Co-occur. RD & MD (*n* = 127)	RD-only (*n* = 74)	MD-only (*n* = 27)
Age at entry, *mean* (*SD*)	6.61 (0.52)	6.53 (0.46)	6.84 (0.58)	6.49 (0.50)	6.68 (0.45)
*Participant characteristics controlled for in analyses*					
IQ, *mean* (*SD*)	91.0 (11.0)	95.3 (11.1)	84.4 (9.3)	90.2 (8.2)	87.9 (8.1)
Lang.: Eng. Only, a *n* (%)	250 (50.2)	131 (48.5)	67 (52.8)	44 (59.5)	8 (29.6)
SES: Low, b *n* (%)	444 (89.2)	229 (84.8)	121 (95.3)	70 (94.6)	24 (88.9)
Sex: Female, *n* (%)	267 (53.6)	152 (56.3)	63 (49.6)	38 (51.4)	14 (51.9)
Ethn.: Hispanic, *n* (%)	242 (48.6)	125 (46.3)	63 (49.6)	36 (48.6)	18 (66.7)
Race: AA/Black, *n* (%)	153 (30.7)	74 (27.4)	48 (37.8)	26 (35.1)	5 (18.5)
Race: White, *n* (%)	125 (25.1)	64 (23.7)	28 (22.0)	21 (28.4)	12 (44.4)
Intervention^c^, *n* (%)	108 (21.7)	21 (7.8)	47 (37.0)	36 (48.6)	4 (14.8)
*Classification measures* ^d^					
TOWRE-2 Index, *mean* (*SD*)	88.3 (15.6)	96.9 (13.7)	74.2 (9.9)	79.1 (7.7)	95.5 (7.8)
GORT Index, *mean* (*SD*)	83.3 (11.7)	89.6 (10.3)	72.6 (8.0)	77.9 (7.3)	86.6 (5.4)
WJ Read. Broad, *mean* (*SD*)	92.6 (16.1)	101.5 (12.9)	77.1 (12.9)	86.4 (9.8)	97.5 (5.4)
WJ Math Broad, *mean* (*SD*)	90.5 (15.0)	98.6 (12.6)	74.6 (10.3)	91.7 (6.8)	82.1 (7.1)

*Note*. RD = reading disability; MD = mathematics disability; Eng. = English; AA = African American; TOWRE = Test of Word Reading Efficiency–Second Edition; GORT = Gray Oral Reading Test; WJ = Woodcock-Johnson III Tests of Achievement; Read. Broad = Reading Broad subtest; Math Broad = Mathematics Broad subtest.

a Participants with no evidence of consistent exposure to a non-English language at home. ^b^Participants whose families reported qualifying for government assistance. ^c^Any measurement points after a participant received reading intervention. ^d^Group means calculated with participant performance averaged across all 5 years of measurement.

### Classification Procedure

The classification procedure utilized both low achievement and persistence as criteria. Participants were classified as RD or MD if they scored below the assigned cut score in two or more school years. For group classification, we used standardized composite scores from four industry-standard assessment batteries that are commonly deployed in research and clinical settings to evaluate broad reading and math achievement. The three reading batteries (Test of Word Reading Efficiency-Second Edition [TOWRE-2], Gray Oral Reading Test [GORT], and Woodcock-Johnson III Tests [WJ-III] Broad Reading) and one math battery (WJ-III Broad Math) are described in the following sections:

#### Assessment Batteries Used for Classification

##### TOWRE Index Standard Score

TOWRE-2 (Torgesen et al., 2012) is a norm-referenced measure that assesses two reading sub-skills via two timed tests: sight word efficiency (real words) and phonemic decoding efficiency (pronounceable nonwords). In each test, examinees are given 45 seconds to read aloud as many presented items as they can. The TOWRE was administered by a trained psychometrist who followed testing procedures outlined in the assessment manual. The TOWRE Index Score incorporates performance on both subtests and is a norm-referenced standardized score. TOWRE Index has a reliability coefficient of .92 (Tarar et al., 2015).

##### GORT Oral Reading Index Standard Score

GORT-5 (Wiederhold & Bryant, 2012) is a measure of oral reading. It has four components, administered by having the examinee read a story aloud: rate (the amount of time taken to read story aloud), accuracy (the number of words pronounced correctly when reading), fluency (a value that combines rate and accuracy results), and comprehension (the number of questions about the story answered correctly). The GORT was administered by a trained psychometrist who followed testing procedures outlined in the assessment manual. The Oral Reading Index Score is norm-referenced, standardized, and accounts for performance on all measured skills. At all age intervals, the Oral Reading Index reliability coefficients exceed .90 (Hall & Tannebaum, 2013).

##### WJ-III Broad Reading and Broad Math Scores

WJ-III Tests of Achievement (WJ-III ACH, Woodcock et al., 2001) is a norm-referenced assessment battery that includes measures of reading, math, writing, and other academic skills. Each subtest of the WJ-III measures a domain-specific narrow ability that is psychometrically defined. For the present study, only the reading and math assessments were administered. Each test was administered by a trained psychometrist who followed testing procedures outlined in the assessment manual.

###### Broad Reading Score

This is a broad measure of reading achievement which clusters performance on key reading skills to create a composite, standardized score. Three reading subtests contribute to this score: letter-word identification (a measure of reading decoding), reading fluency (a measure of reading speed and semantic processing speed), and passage comprehension (a measure of reading comprehension and cloze ability). As reported in the test manual, across ages 5–19 years, the median reliability coefficient for Broad Reading is .93.

###### Broad Math Score

This is a broad measure of math achievement which clusters performance on key math skills to create a composite, standardized score. Three math subtests contribute to this score: calculation (a measure of math achievement and calculation procedures), math fluency (a measure of arithmetic automaticity), and applied problems (a measure of quantitative reasoning and applied math knowledge). As reported in the test manual, across ages 5–19 years, the median reliability coefficient for Broad Math is .95.

#### Operational Definition of Persistent Low Achievement

##### Low Achievement Cut Scores

Participants were flagged for low reading achievement if, at any single measurement point, their standardized composite scores on two out of three of the reading batteries (TOWRE, GORT, & WJ-III Broad Reading) were lower than 85, which is one standard deviation below the mean and around the 16th percentile. Although there are no universally agreed-upon classificational criteria for RD, this cut score is common in both the RD literature and clinical practice (Hammill & Allen, 2020). Requiring evidence of low achievement on two out of three assessments provides some protection against misclassification errors.

Participants were flagged for low math achievement if their standardized composite score on the math assessment battery (WJ-III Broad Math) was lower than 85, one standard deviation below the mean. Cut scores for MD classification vary widely in both the literature and in clinical practice, ranging from the 5th to the 35th percentile (Murphy et al., 2007). A math cut score of 85, around the 16th percentile, is near the middle of that range and has the added benefit of being consistent with the reading cut score.

##### Persistence Criteria

Participants were classified in our sample as RD or MD only if their performance met the above-described low achievement criteria for more than one school year. Including persistence as a criterion for classification of learning disability is common in the MD literature and is recommended to protect against over-classification error (Murphy et al., 2007).

For math data, which was collected annually, participants were classified as MD if they scored below the defined cut score at two of their measurement points. The same principle was used for the semi-annual reading data, with the added requirement that the two measurement points at which the participant scored below the cut score were in two different grade levels—meaning a participant was not classified as RD if their low performance was limited to a single school year, even if both measurements during that school year were below the cut score.

We did not constrain the grade range used for persistence criteria, thus a below-cutoff achievement in any 2 years of the 5-year study could be counted toward persistence. The purpose of this was to increase the likelihood of capturing borderline cases. An unfortunate byproduct of using cutoff scores is that if a participant’s performance regularly hovers around the cutoff score, it will sometimes land just above, other times just below. Participants in this liminal space functionally exhibit the impairments associated with learning disability, even if their scores do not consistently characterize them as such. Educators and clinicians tend to use professional judgment to identify disability in borderline cases like these. Our longitudinally broad-persistence criteria improve the chances that our fixed procedure catches the types of cases that educators and clinicians are likely to catch in a shorter time frame with professional judgment. About 45% of the disability-classified participants had already met persistence criteria by Grade 2. By Grade 3, it was 70%, and by Grade 4, it was 95%.

#### Verification of Group Separation

Table 1 reports group means and standard deviations on the four assessment batteries used to classify disability groups. To verify that our classification procedure resulted in expected separation between groups on affected and unaffected skills, we conducted one-way ANOVAs and follow-up pairwise comparisons between groups on the four assessment batteries used for classification. One challenge of using longitudinally broad persistence criteria is that there is no single time point to run comparative analyses with for verification of group separation. For each assessment battery, instead of using a single time point, we calculated each participant’s average performance across all 5 years of measurement and used these averages for group comparisons.

As expected, the four-group ANOVAs showed significant group differences on all assessment batteries used for classification: TOWRE—*F*(3, 488) = 127.250, *p* < .001; GORT—*F*(3, 488) = 109.942, *p* < .001; WJ-III ACH Broad Reading—*F*(3, 482) = 122.047, *p* < .001; and Broad Math—*F*(3, 488) = 141.122, *p* < .001. Follow-up pairwise comparisons showed that disability groups differed from each other in expected ways on each assessment battery. On all three reading batteries, each of the groups with RD (i.e., the Co-occurring and RD-Only groups) show significantly lower scores than each of the groups without RD (i.e., the No Disability and MD-Only groups), with Bonferroni adjusted *p* values below .0001 for each comparison. Likewise, on the math battery, both groups with MD show significantly lower scores than both groups without MD, with adjusted *p* values below .001 for each comparison.

Pairwise comparisons showed additional group differences beyond the expected ones. On each reading battery, the Co-occurring group showed lower levels than the RD-Only group, with varying levels of effect for each reading battery (TOWRE *p* = .026; GORT *p* = .0004; WJ-III Broad Read *p* < .00001). Similarly, on the WJ-III Broad Math battery, the Co-occurring group was lower than the MD-Only group (*p* = .008). On this math battery, an additional difference was found between the No Disability group and the RD-Only group (*p* < .0001), indicating that although the RD-Only group’s math performance meaningfully exceeds that of the two groups with MD, it is lower than that of their non-disabled peers.

### Variables

#### Reading and Math Subtests Used as Outcome Measures

To measure reading and math skill growth over time, we utilized W scores from seven subtests of WJ-III ACH (Woodcock et al., 2001) as outcome variables. Each subtest measures a domain-specific narrow ability that is psychometrically defined. W scores are mathematically transformed raw scores that are well-suited for statistical analysis of skill growth in multiple domains because they are change-sensitive equal-interval scales that enable comparisons across different ages, achievement levels, and tests (Benson et al., 2018; Jaffe, 2009). The seven relevant subtests (four reading and three math) are briefly described below. Each test was administered by trained psychometrists who followed testing procedures outlined in the assessment manual.

##### Letter Word ID

Examinees read single words of increasing difficulty aloud until they produce six consecutive errors or complete all test items. Words are presented in isolation, rather than in context. As reported in the test manual, Letter Word ID has a median reliability of .91 across ages 5–19 years.

##### Word Attack

Examinees decode increasingly difficult low-frequency words and pseudowords until they produce six consecutive errors or complete all test items. It has a median reliability of .87 across ages 5–19 years.

##### Reading Fluency

Examinees are given 3 minutes to read a series of simple sentences and state whether the sentences are true or false. It has a median reliability of .90 across ages 5–19 years.

##### Passage Comprehension

Examinees complete several different types of test items, some involving picture-symbol matching, and others involving cloze exercises wherein they select words to fill blank spaces in sentences and paragraphs of increasing complexity, until they produce six consecutive errors or complete all test items. It has a median reliability of .83 across ages 5–19 years.

##### Calculation

Examinees perform math operations of increasing difficulty, beginning with simple number writing, and continuing into geometry, trigonometry, and calculus operations, until they produce six consecutive errors or complete all test items. All numbers are represented as Arabic numerals—no verbal representations of numbers are used in either written or spoken form. Calculation has a median reliability of .85 across ages 5–19 years.

##### Math Fluency

Examinees are given 3 minutes to answer as many basic computation questions as they can. These basic computations are single-digit addition, subtraction, and multiplication. All numbers are represented as Arabic numerals—no verbal representations of numbers are used in either written or spoken form. Math Fluency has a median reliability of .89 across ages 5–19 years.

##### Applied Problems

Examinees are orally presented with contextualized math problems which may include relevant and irrelevant information, then they are instructed to select and perform the appropriate calculations to solve each problem, until they produce six consecutive errors or complete all test items. This test includes a variety of numerical representations, including Arabic digits, spoken and written number words, and non-symbolic magnitude representations. Applied Problems has a median reliability of .92 across ages 5–19 years.

#### Demographic and Other Variables—Covariates

All categorical demographic variables reported in the sample description (see also Table 1) were included as binary variables in our models. These variables were included primarily for control purposes and are referred to as covariates.

##### Language

This variable approximates multi-language exposure. We utilized several avenues (language of communication with participant’s parents, participant answering assessment questions in a non-English language or otherwise showing preference for another language, etc.) to seek evidence that a participant has consistent or daily exposure to a language other than English. For this variable, participants for whom there was no evidence of meaningful consistent non-English exposure were coded as the reference group.

##### Ethnicity and Race

Upon entry into the study, parents/guardians were invited to report their participating child’s race and ethnicity. They were able to select as many race and ethnicity categories as apply. Participants identifying exclusively as Asian (*n* = 6), American Indian or Alaska Native (*n* = 0), or Native Hawaiian or Other Pacific Islander (*n* = 6) were not represented in high-enough frequency in our sample to include as separate variables in our analyses. Three variables were created for Hispanic ethnic identity (with those identifying as Hispanic coded as the reference group and assigned a value of 1), Black racial identity (with those identifying as Black or African American assigned a value of 1, all others 0), and White racial identity (with those identifying as White or Caucasian assigned a value of 1, all others 0). As these were non-exclusive, a single participant could reasonably have a value of 1 in all three variables. Participants with a value of 0 on all three variables either opted not to report their ethnic/racial identity or did not identify as Hispanic, Black, or White.

##### Socioeconomic Status

Upon entry into the study, parents/guardians were invited to report, via questionnaire, whether their family qualified for and received government assistance at the federal, state, or local level. Examples of government assistance included Temporary Assistance for Needy Families (TANF); Special Supplemental Nutrition Program for Women, Infants, and Children (WIC); and Food Stamps. Participants whose families reported having received no government assistance were coded as the reference group.

##### Sex

Participant sex was reported by parents/guardians upon study entry. Female was coded as the reference for the sex variable.

##### IQ

Participant IQ was measured with the PRI standard score of the WASI-II (Wechsler, 2011). Since IQ was solely used as a covariate, it was grand-mean centered at zero before including in the analysis to allow our group-based contrast coefficients to be interpreted correctly and in their proper scale.

##### Reading Intervention Completion

Part of the NHLP study design included the implementation of intervention programs in some schools. Intervention is not a focus of the present analyses; however, given the likelihood of intervention impacting the growth patterns of those involved, a time-varying intervention variable was included in our analyses, in which a value of 1 was associated with any data point after a participant had received the Phonological and Strategy Training (PHAST) intervention (Lovett et al., 2000). Any data point before intervention, or any data point associated with participants who did not receive PHAST, was marked with a 0.

#### Time Variable

Although psychometric data were collected in a roughly patterned way in fall and spring semesters, logistical constraints made it impossible to gather data at equidistant intervals. To account for unequal testing intervals, we created a Time variable to use in all models that represents the elapsed time (in months) between a participant’s start date at the beginning of Grade 1 and each subsequent measurement point thereafter. Each participant’s first measurement in the Fall of Grade 1 was designated as their start date and given a time value of zero. Participants who enrolled after Grade 1 were assigned a pseudo-start date based on the average start date of those with existing entry data—this ensured that the time variable for all participants roughly reflected the timeline of progression from early Grade 1 through early Grade 5. We did not use participant age as a covariate in our growth models because it would be too highly correlated with our time variable, and because our sample has minimal age variability.

### Analysis Procedure

We utilized R statistical software (R Core Team, 2017), R Studio (RStudio Team, 2020), and ggplot2 (Wickham, 2016) for data preparation, as well as pre- and post-analysis data visualization. For our statistical models, we utilized SPSS 27 (IBM Corp., 2020). To look for outliers and violations of assumptions, we visually inspected individual participant growth plots (graphed in groups of 20–25 participants using ggplot2) on each outcome variable.

As is common in longitudinal datasets, missing data occurred due to attrition, late entry, and missed assessments. On average, 66%–76% of our participants were represented at any given measurement point, with the exception of the final two measurement points, which averaged 34%–38% due to the preclusion of second-cohort data collection in 2020. Our mixed-effects models, described in greater detail below, are robust to missing data. We used the direct Maximum Likelihood (ML) approach, which utilizes all available observations over time, assigning greater weight to cases with more timepoints. Thus, timepoints that have more data density are estimated with greater precision (Curran et al., 2010). Nevertheless, to double-check that data missingness was not systematically related to participant characteristics, participants with nine complete observations over the 5-year span (*n* = 106) were compared to those missing data at any time point. The results suggest there were no meaningful differences between these two groups on basic demographics.

For our primary analyses, we used disability group membership (MD-only, RD-only, MD & RD, and No Disability) to create three dummy coded variables, each centered around 0. These three variables were created to facilitate independent orthogonal comparisons that addressed our three research questions described in the following paragraphs:

For Research Question 1 (*Are the math and reading growth trajectories of children with learning disabilities similar to those of children without disabilities?*), the Any Disability variable was created, which compares participants with any disability (co-occurring or single) to those without disability. Assigned values: MD-only (−0.25), RD-only (−0.25), or MD & RD (−0.25), No Disability (0.75)

For Research Question 2 (*Are the growth trajectories of children with co-occurring MD and RD similar to those of children with single-domain disabilities?*), the Co-occurring Disability variable was created, which compares participants with co-occurring MD and RD disability to those with single disability in either domain. Assigned values: MD-only (0.25), RD-only (0.25), or MD & RD (−0.5), No Disability (0)

For Research Question 3 (*Do children with single MD and single RD show varying degrees of specificity in their developmental patterns of impairment?*), the Single Disability Type variable was created, which compares participants with single MD to those with single RD. Assigned values: MD-only (0.25), RD-only (−0.25), MD & RD (0), or No Disability (0)

We used linear mixed-effects models to account for variability at both the individual and group level, modeling between-individual differences in within-individual change (Nesselroade, 1991). We built seven three-level nested models, one for each outcome variable. Our structure was that of individual change (Level 1) nested within participants (Level 2), nested within schools (Level 3). Due to the large sample size, we utilized ML estimation in each model. Likewise, with unequally spaced test intervals, and sufficient sample size, we opted for the atheoretical unstructured covariance matrix (UN).

For each of our seven outcome variables, we followed best practices in building models as described by Singer and Willett (2003), beginning with an unconditional means model wherein the intercept, and nothing else, was included in both fixed and random effects. When we affirmed proper estimation of the means model, we built an unconditional growth model by adding a linear growth term to the participant-level random effects. As the school level of nesting was not of particular interest to our interpretations, we included only intercept and no time terms in the school-level random effects of all models. Next, we structured an additional growth model with a quadratic growth term added in addition to linear growth in the random effects. If the addition of quadratic growth did not produce better model fit—operationalized as lower Akaike information criterion (AIC) and Bayesian information criterion (BIC) values than models with linear growth alone—or resulted in model nonconvergence, quadratic growth was removed from random effects to maintain the most parsimonious random-effects structure. This was the case with two of our seven outcome variables (Word Attack and Applied Problems).

After establishing random-effects structures that balanced parsimony and goodness of fit, we then structured fixed effects, starting with linear growth then adding quadratic growth terms to fixed effects. In all cases, both linear and quadratic growth terms reached significance and were left in the model. All covariates—IQ, Non-English Language Exposure, SES, Sex, Ethnicity (Hispanic), Race (AA/Black), Race (White), and Intervention—were added to fixed effects simultaneously. Next, covariate-by-linear growth interaction terms were added to fixed effects, and only the interaction terms that reached significance—defined as *p* < .05—remained in each model to maintain model parsimony.

Having established random effects and best-fitting growth and covariate structures for fixed effects, our final step in model building was inserting our predictors of interest—our three disability grouping variables: Any Disability, Co-occurring Disability, and Single Disability Type. All three grouping variables were entered simultaneously, along with disability group-by-linear growth interaction terms for each variable.

## Results

**Figure 1.**
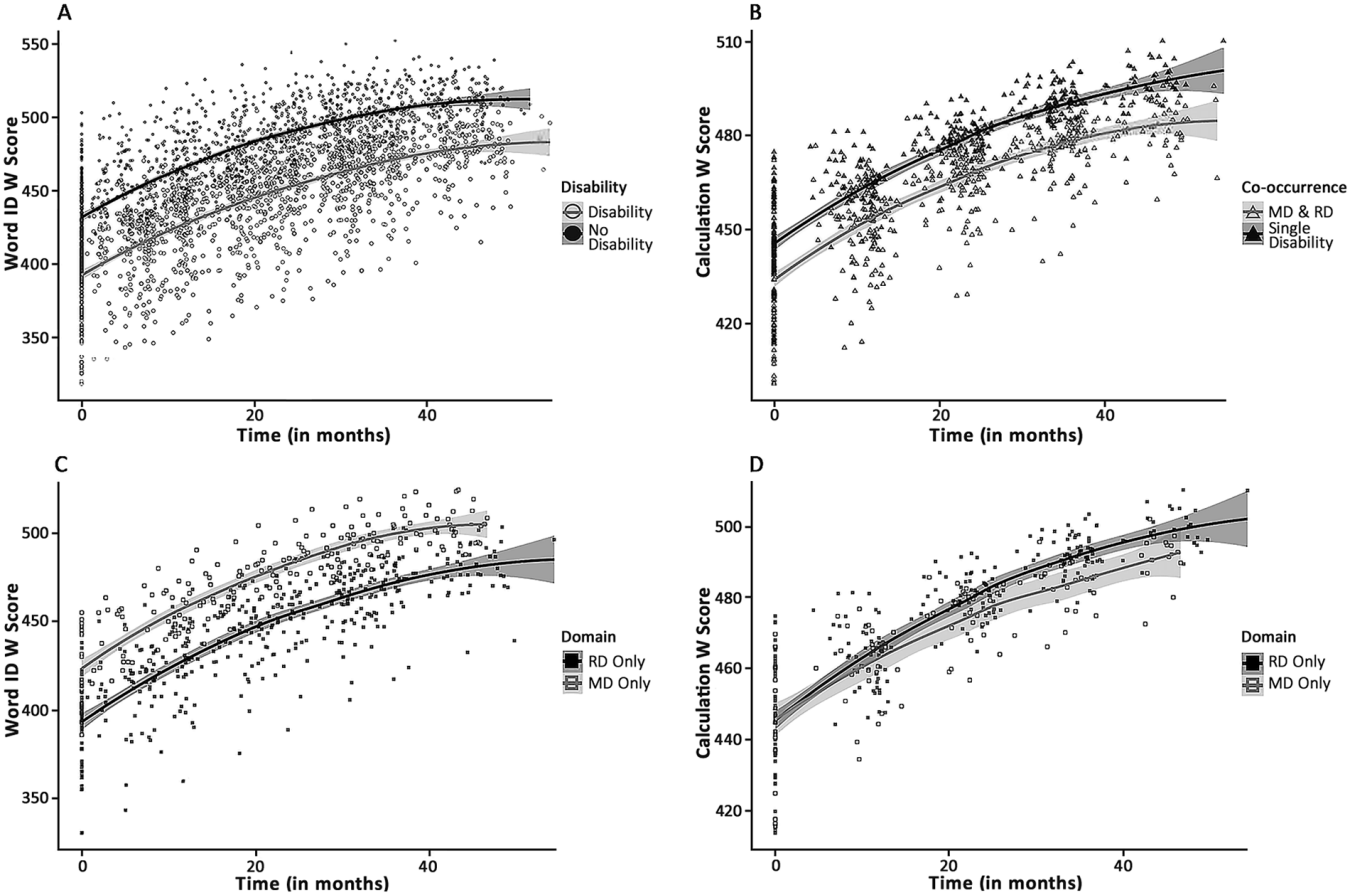
Parallel growth trajectories at disparate skill levels evident between groups with and without disability (1A) and groups with co-occurring MD & RD versus single disability in either domain (1B). Word ID and Calculation outcomes shown as representative examples of the patterns seen across most variables (see exceptions in Figure 2). Imbalanced impairment specificity evident in single disability groups, where MD-only and RD-only groups show disparate levels of reading skill (1C, in Word ID), but similar levels of math skill (1D, in Calculation). Plots show model predicted W scores plotted over time (in months), visualized in R using ggplot2 with loess smoothing for regression lines. Y-axes automatically scaled to reflect data range of each plotted outcome. Shaded areas represent 95% confidence intervals. *Note*. MD = mathematics disability; RD = reading disability; Word ID and Calculation are subtests of the Woodcock-Johnson III Tests of Achievement.

Table 2 shows the random effects of all reading and math outcomes. Tables 3 and 4 show fixed-effects estimates and standard errors for all base, covariate, and predictor variables in reading and math outcomes. Significant fixed effects of intercept, linear growth, and quadratic growth were evident in all seven outcome variables. The negative coefficient for quadratic growth indicated that as time progressed, the rate of improvement for each skill slowed (see Figure 1).

**Table 2.** Random-Effects Covariance Parameter Estimates.

WJ-III measure	Parameter											
	Intercept			Linear growth			Quadratic growth			Intercept: School		
	Est.	*SE*	*p*	Est.	*SE*	*p*	Est.	*SE*	*p*	Est.	*SE*	*p*
Reading variables												
Word ID	710	59	<.001	1.61	0.21	<.001	0.00036	0.00007	<.001	17.54	13.49	.194
Word attack	303	26	<.001	0.17	0.02	<.001	—	—	—	6.5	5.75	.259
Reading fluency	43.3	4.4	<.001	0.19	0.03	<.001	0.00007	0.000012	<.001	2.32	1.5	.127
Passage comp.	332	29	<.001	0.93	0.12	<.001	0.00023	0.00004	<.001	3.18	2.9	.289
Math variables												
Calculation	221	23	<.001	0.63	0.13	<.001	0.00017	0.00005	.001	9.87	5.67	.082
Math fluency	5.7	0.85	<.001	0.02	0.007	.001	0.000006	0.000003	.054	1.36	0.73	.064
Applied problems	145	15	<.001	0.11	0.02	<.001	—	—	—	24.73	15.22	.104

*Note.* Blank cells indicate where quadratic growth was not part of the optimized random-effects growth model; WJ-III = Woodcock-Johnson III Tests of Achievement; comp. = comprehension.

**Table 3.** Fixed Effects for WJ-III Reading Subtest W Scores.

Source	Word ID		Word attack		Reading fluency		Passage comp.	
	Est.	*SE*	Est.	*SE*	Est.	*SE*	Est.	*SE*
Intercept	411***	3.6	450***	2.4	467***	1.1	434***	2.1
Linear growth	3.2***	0.09	2.1***	0.06	0.71***	0.04	2.0***	0.07
Quadratic growth	–0.03***	0.002	–0.03***	0.001	–0.004***	0.0007	–0.02***	0.001
Covariate main effects								
IQ	0.35**	0.13	0.30**	0.10	0.08*	0.03	0.25***	0.06
Language	–2.4	2.5	–1.3	1.8	–0.22	0.82	0.78	1.5
SES	6.2*	3.2	3.2	2.3	0.79	1.1	5.0*	2.0
Sex	2.8	1.8	0.81	1.3	1.5*	0.67	3.7**	1.2
Ethnicity: Hisp.	–13***	3.0	–3.8*	1.7	–2.8***	0.78	–3.2*	1.5
Race: AA/Black	1.2	2.5	–0.52	1.8	0.16	0.84	2.1	1.6
Race: White	5.1*	2.3	2.1	1.7	1.4	0.76	1.3	1.5
Intervention	–0.97	1.3	9.6***	2.5	1.9***	0.51	–0.86	0.91
Covariate-by-linear growth interactions								
IQ × Linear	–0.004	0.003	–0.005	0.003	—	—	—	—
Language × Linear	—	—	—	—	—	—	—	—
SES × Linear	—	—	—	—	0.10**	0.04	—	—
Sex × Linear	—	—	—	—	0.05*	0.02	—	—
Ethnicity × Linear	0.21***	0.06	—	—	—	—	—	—
Intervention × Linear	—	—	–0.13	0.09	—	—	—	—
Disability group main effects								
Any disability	25***	3.1	17***	2.2	7.6***	0.79	13***	1.9
Co-occurring disability	27***	5.3	13**	3.8	6.1***	1.3	16***	3.3
Single disability type	64***	12.3	37***	8.9	15***	3.1	37***	7.7
Disability group-by-linear growth interactions								
Any disability × Linear	–0.09	0.07	–0.07	0.06	0.005	0.03	–0.04	0.05
Co-occurring disability × Linear	–0.11	0.11	0.08	0.10	0.06	0.04	–0.16*	0.08
Single disability type × Linear	–0.52	0.27	–0.04	0.24	–0.02	0.10	–0.67**	0.19

*Note.* Blank cells indicate where Covariate × Linear interactions were not included in models. WJ-III = Woodcock-Johnson III Tests of Achievement; Comp. = comprehension; AA = African American.

* *p* < .05, **p < .01, ***p < .001.

**Table 4.** Fixed Effects for WJ-III Math Subtest W Scores.

Source	Calculation		Math fluency		Applied problems	
	Est.	*SE*	Est.	*SE*	Est.	*SE*
Intercept	449***	2.1	483***	0.60	439***	2.5
Linear growth	1.6***	0.07	0.33***	0.02	1.1***	0.06
Quadratic growth	–0.01***	0.001	–0.003***	0.0003	–0.004***	0.001
Covariate main effects						
IQ	0.26***	0.05	0.03	0.02	0.33***	0.07
Language	–8.1***	1.8	–1.6***	0.43	3.0	1.6
SES	1.8	1.7	0.38	0.49	3.0	2.0
Sex	–0.04	0.97	–0.38	0.28	–0.68	1.2
Ethnicity: Hisp.	0.18	1.2	–0.73*	0.37	–1.9	1.5
Race: AA/Black	1.2	1.4	–0.26	0.40	0.31	1.7
Race: White	–0.15	1.2	0.37	0.36	2.0	1.5
Intervention	1.4	1.1	0.81*	0.36	1.5	1.3
Covariate-by-linear growth interactions						
IQ × Linear	—	—	0.0002	0.0007	0.002	0.002
Language × Linear	0.24***	0.04	0.04**	0.01	—	—
SES × Linear	—	—	—	—	—	—
Sex × Linear	—	—	—	—	—	—
Ethnicity × Linear	—	—	—	—	—	—
Intervention × Linear	—	—	—	—	—	—
Disability group main effects						
Any disability	8.1***	1.7	1.8***	0.38	7.0***	1.7
Co-occurring disability	10**	3.0	1.5*	0.65	4.2	2.9
Single disability type	–8.6	7.1	–1.3	1.5	–11	6.9
Disability group-by-linear growth interactions						
Any disability × Linear	0.08	0.05	0.05**	0.02	0.28***	0.06
Co-occurring disability × Linear	0.05	0.08	0.04	0.03	0.16	0.09
Single disability type × Linear	–0.13	0.20	–0.14*	0.06	–0.71**	0.22

*Note.* Blank cells indicate where Covariate × Linear interactions were not included in models. WJ-III = Woodcock-Johnson III Tests of Achievement; Hisp. = Hispanic; AA = African American.

* *p* < .05, **p < .01, ***p < .001.

### Covariates

Covariate fixed effects are shown in Tables 3 and 4. A higher IQ was reliably associated with higher scores on six of the seven outcome variables: Word ID, Word Attack, Reading Fluency, Passage Comprehension, Calculation, and Applied Problems.

The SES covariate was positively associated with Word ID and Passage Comprehension, such that those children, whose family did not indicate having received government assistance, tended to score higher on those outcomes. Sex was significantly related to Reading Fluency and Passage Comprehension, with females scoring higher on both outcomes.

White racial identity was associated with higher scores on Word ID, and a significant Ethnicity effect was evident in all reading outcomes (see Table 2) and Math Fluency (see Table 3), with those identifying as Hispanic tending to show lower scores on all five outcomes. The Language covariate was significantly related to Calculation and Math Fluency, with a negative coefficient in both cases, indicating that English-only children scored lower on these two math outcomes than children who had evidence of everyday exposure to a non-English language.

Intervention completion was associated with higher scores on Word Attack, Reading Fluency, and Math Fluency at post-intervention testing points than pre-intervention testing points among intervention recipients and all testing points among those who did not receive intervention.

Five covariate-by-linear growth interactions met significance: three for reading (see Table 3) and two for math (see Table 4). An ethnicity-by-linear growth interaction appeared in Word ID, in which an early score gap, wherein non-Hispanic participants scored slightly higher than Hispanic participants, narrowed and disappeared entirely over time. In Reading Fluency, there were significant interactions for SES-by-linear growth and sex-by-linear growth. In both cases, a score gap increased over time, with females outscoring and outpacing their non-female peers in Reading Fluency growth, and children with low-SES markers showing slightly less steep growth over time than their peers. For math, language-by-linear growth interactions were evident in Calculation and Math Fluency. In both cases, an early score gap in which multi-language children outperformed their English-only peers reduced over time, leaving both language groups at similar levels around Grade 4.

### Disability Group Differences

#### Research Question 1—Longitudinal Parallels With and Without Disability

**Figure 2.**
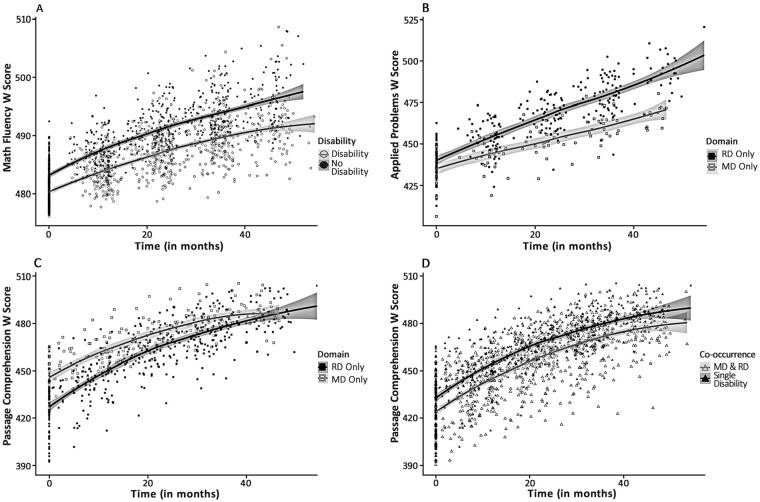
Exceptions to general patterns of parallel growth across disability groups evidenced in unique examples of diverging and converging skill growth. Examples include a subtle widening of the skill gap in Math Fluency between groups with and without disability (2A), steeper growth in Applied Problems skill in RD-only compared to MD-only groups (2B), disappearance over time of a Passage Comprehension skill gap between RD-only and MD-only groups (2C), and a subtle lessening of the skill gap in Passage Comprehension between groups with co-occurring versus single disabilities (2D). Plots show model predicted W scores plotted over time (in months), visualized in R using ggplot2 with loess smoothing for regression lines. Y-axes automatically scaled to reflect data range of each plotted outcome. Shaded areas represent 95% confidence intervals. *Note*. MD = mathematics disability; RD = reading disability; Math Fluency, Applied Problems, and Passage Comprehension are subtests of the Woodcock-Johnson III Tests of Achievement.

Disability classification, regardless of type, was associated with lower scores on all outcome variables (see Figure 1A and Supplemental Figure S1). The rate of growth between participants with and without any disability was the same for all reading assessments and calculation. The score gap for Math Fluency and Applied Problems widened over time (see Figure 2A and Supplemental Figure S1G).

#### Research Question 2—Pattern Similarities in Co-Occurring and Single Disabilities

The rate of growth between participants with co-occurring versus single disability was the same for all variables except Passage Comprehension. In Passage Comprehension, the combined single-disability group had a slightly higher rate of growth (see Figure 2D). In all outcomes apart from Applied Problems, the combined single-disability group (which averaged MD-only and RD-only performance) had higher achievement levels.

Due to limited interpretability of our primary comparison which combined the two single-disability groups, we performed post-hoc contrasts to provide more useful details about co-occurrence. In two contrasts, the Co-occurring MD and RD group was compared separately to the MD-only group and the RD-only group. Growth model construction for these contrasts followed the same procedure described for the primary analyses. With the Co-occurring group included in each contrast, these post-hoc analyses do not have the same level of independence as our primary analysis, but they add valuable domain-specific interpretability as a tradeoff. The results were as follows:

The MD-only group outperformed the Co-occurring group on all reading outcomes (all *p*’s < .0001), and the RD-only group outperformed the Co-occurring group on all math outcomes. On all four reading outcomes, the RD-only group did not differ from the Co-occurring group in performance level or growth rate. Similarly, on all three math outcomes, the MD-only group did not differ from the Co-occurring group in performance level or growth rate. A few cross-domain effects were evident in growth rates. On Passage Comprehension, the Co-occurring group showed faster growth than the MD-only group (*b* = .24, *SE* = .10, *p* = .017). By contrast, the RD-only group showed faster growth than the Co-occurring group on both Math Fluency (*b* = .06, *SE* = .02, *p* = .005) and Applied Problems (*b* = .30, *SE* = .07, *p* < .001).

#### Research Question 3—Varying Specificity in MD and RD Impairments

Score differences based on single-disability type were evident in all the reading variables but none of the math variables (see Figure 1C and 1D and Supplemental Figure S3). In all four reading variables, the MD-only group scored higher than the RD-only group (see Figure 1C). In contrast, none of the math variables showed significant score differences between disability types—RD-only and MD-only groups scored comparably on math outcomes (see Figure 1D).

Three variables, Passage Comprehension, Math Fluency, and Applied Problems, showed significant differences in growth between MD-only and RD-only groups. In Math Fluency and Applied Problems, the pattern showed a widening score gap between the two groups, with RD-only participants improving at a faster rate than MD-only participants (see Figure 2B and Supplemental Figure S3F). The inverse was evident in Passage Comprehension, as an early score gap narrowed over time with MD-only participants outperforming their RD-only peers (see Figure 2C).

### Secondary Analyses With More Conservative MD Cut Scores

A secondary analysis was conducted with a more conservative cut score for MD classification, to verify that the pattern of results remains even when the MD cut score is modified to be more closely aligned with the math literature. This cut score was 76.3, determined by subtracting the standard error of the WJ Broad Math score (3.67, as reported in the Schrank et al., 2001 WJ III Technical Abstract) from 80, the standard score representing the 10th percentile. The 10th percentile has been recommended in the math learning disability literature as an efficient and balanced cutoff (Geary, 2011; Geary et al., 2007; Murphy et al., 2007). We subtracted the standard error as an extra protection against overclassification. With the new classification criteria, the distribution of disability group membership changed to the following: RD-only (*n* = 134), MD-only (*n* = 7), MD & RD (*n* = 67), and No Disability (*n* = 290).

With three exceptions, the pattern of results in the secondary analyses matched those of the primary analyses. With a more conservative MD cut score, the relation between Math Fluency and IQ became significant (*b* = 0.04, *t*(394) = 2.3, *p* = .02). In Passage Comprehension, the primary analyses showed a growth rate difference between participants with co-occurring versus single disabilities—this difference was not evident with the more conservative cut score (*b* = −0.12, *t*(250) = −1.0, *p* = .310). Lastly, the Applied Problems growth rate differences between MD-only and RD-only children in the primary analyses did not reach significance in the secondary analyses (*b* = −0.62, *t*(254) = −1.7, *p* = .094). This last result is likely due to the loss of power created when the change in MD cut score decreased the number of participants in the MD-only group from 27 to 7.

## Discussion

In this study, we utilized linear mixed-effect models to investigate patterns of math and reading subskill growth in children with and without MD and RD. We conducted planned orthogonal comparisons and post-hoc contrasts to observe group differences on seven domain-specific reading and math outcomes. Our analyses were framed around three research questions, each discussed in the following sections:

### Research Question 1—Longitudinal Parallels With and Without Disability

Our first research question asked, “Are the math and reading growth trajectories of children with learning disabilities similar to those of children without disabilities?” To address this, we compared children with any evidence of MD and/or RD to those without. Our findings suggest that, with few exceptions, the answer to this question is yes. Children with and without learning disabilities do not appear to differ in their rate and shape of skill growth across Grades 1–5. Both groups show similar shape patterns in the way their skills develop over time, exhibiting steady improvement in the early grades in most cases followed by a gradual deceleration of growth as time progresses, a pattern visible in Figure 1A.

This finding has several implications. First, this suggests that developmental change in foundational reading and math skills follows a largely predictable pattern of change over time in children with and without disabilities, regardless of early skill level. Second, this verifies that disability is not a matter of simple developmental delay in skill acquisition. Were it a matter of delay, we would expect to see the growth trajectories of children with and without disabilities converge over time. Instead, we see that reading and math skills in children with disabilities grow and plateau at a rate, and in a developmental time frame, almost identical to children without disabilities, they simply grow and plateau at lower skill levels. This means that early skill difficulties in children with disabilities do not naturally resolve over time but necessitate targeted intervention.

From an implementation perspective, this finding underscores the importance of early identification and intervention, since significant disability-related impairments were already present by the start of Grade 1. From a research perspective, this highlights a particular difficulty in the study of developmental learning disabilities—that the underlying impairments associated with learning disabilities begin to hinder skill development well before the impacted skills can be reliably and meaningfully assessed. By the time we can accurately assess math and reading skills, the impairments associated with learning disabilities will have already created skill disparities that are large enough to require concerted and targeted work to overcome. This premise is a primary driver of the larger goal of the NHLP project to identify genetic markers of learning disability that may help identify children at risk of disability long before the phenotype manifests.

There were two exceptions to the pattern described earlier. In the math domain, Math Fluency and Applied Problems revealed growth rate differences between children with and without disability. Figure 2A shows that there was a subtle but significant widening of the skill gap between disability groups in Math Fluency—this is evident in Applied Problems as well. In both cases, it appears that children without disabilities grew in a more consistently linear upward trajectory, whereas children with disabilities showed evidence of flatter growth that subtly decelerated over time. These exceptions appear to have their origins in the distinct RD-related and MD-related growth patterns and will be discussed with those findings.

### Research Question 2—Co-Occurring Disability Versus Single Disability

Our second research question asked, “Are the growth trajectories of children with co-occurring MD and RD similar to those of children with single-domain disabilities?” To address this, we compared children with co-occurring MD and RD to those with a single disability in either. We probed this question further with post-hoc contrasts that compared the Co-occurring group separately to each isolated disability group (MD-only and RD-only). Our findings suggest that, with few exceptions, the answer to this question is yes. Children with co-occurring and single disabilities have consistently similar shapes and rates of growth in math and reading skills. With three exceptions tied to the cross-domain growth differences discussed in the following section, our primary analyses do not reveal differences in growth between the co-occurring and single-disability groups. Post-hoc contrasts confirm this by showing that the *math* trajectories of children with co-occurring MD and RD do not differ from those of the MD-only group. Likewise, their *reading* trajectories do not differ from the RD-only group. These alignments lend the strength of statistical independence from the primary analysis to the more interpretable findings from post-hoc contrasts.

There were three cross-domain growth differences evident in the post-hoc analyses. Each of these differences is a parallel of a distinct MD or RD growth pattern that manifests clearly in the MD-only versus RD-only findings. These patterns and their potential origins and implications are discussed in detail in the next section. Their parallels in the co-occurring disability post-hoc analyses include the following: On Passage Comprehension, the Co-occurring group shows significantly faster growth than the MD-only group, but not the RD-only group. In this skill, children with co-occurring MD and RD and children with single RD share a common Passage Comprehension growth pattern, which is not altered by co-occurrence. In this pattern, children with RD (whether co-occurring or isolated) show low early skill level followed by steady steep growth. In contrast, the MD-only group shows a pattern of higher early skill level followed by shallower growth, similar to the No Disability group. Likewise, in separate comparisons, the RD-only group outpaced the co-occurring MD and RD groups on Math Fluency and Applied Problems growth. On these skills, children with co-occurring MD and RD and children with single MD share common growth patterns, which are not altered by co-occurrence. This pattern is one of low early skill level followed by slow shallow growth. These findings indicate that in Grades 1–5, the growth patterns of co-occurring MD and RD reflect a combination of the math domain patterns of MD and the reading domain patterns of RD, with no evidence of compounding cross-domain impairment effects.

Although our primary analysis shows achievement-level differences between the co-occurring and single-disability groups, this is not a meaningful or accurate finding given that single MD and single RD were averaged in our primary analyses. We relied on post-hoc analyses to conduct separate comparisons between the Co-occurring group and each single-disability group separately. Post-hoc analyses revealed that the achievement levels of the Co-occurring group do not differ from those of the separate MD-only and RD-only groups.

Our findings align with the additive model of co-occurrence, which posits that MD and RD are distinct but related disorders whose co-occurrence can be characterized as the additive result of MD-related difficulties plus RD-related difficulties. The additive model of MD and RD has been well-documented in domain-general cognitive processes (Grant et al., 2020; Moll et al., 2016; Willcutt et al., 2013; Wilson et al., 2015; Wong & Ho, 2021). Our findings further extend support for the model by demonstrating that it manifests in domain-specific skills themselves and maintains consistency over time amid skill growth in Grades 1–5.

### Research Question 3—Single Reading Versus Single Math Disability

Our third research question asked, “Do children with single MD and single RD show varying degrees of specificity in their developmental patterns of impairment?” To address this, we compared children with single MD to those with single RD. Our findings suggest that there are disparate levels of specificity in MD versus RD. These specificity disparities become clear when looking at cross-domain skills. On each of our reading outcomes, MD-only children show achievement levels and growth rates comparable to those of children without disability (see Figure 1C). In contrast, on all three math outcomes, the achievement levels of RD-only children are low enough to be statistically indistinguishable from MD-only children (see Figure 1D). It seems RD-only children experience clear difficulties with fundamental math skills despite having performed well enough in the domain to escape MD classification. Growth patterns in the RD-only group suggest that early difficulties with math fluency and applied problem-solving resolve over time, but difficulties with calculation do not. It is important to note that overrepresentation of children with RD in our sample resulted in a larger group of participants with RD-only (*n* = 74) compared to those with MD-only (*n* = 27). Therefore, patterns observed in the smaller MD-only group may have more limited generalizability than those observed in the larger RD-only group.

#### MD Patterns—Domain Specificity, Low Achievement, Slow Growth

The impairment profile of MD appears to lean heavily toward domain specificity. In the MD-only group, impairments manifest clearly and exclusively in the math domain. On all three math skills, children with MD have low overall achievement, but their impairment is most obvious in their distinctively slow growth. This flat growth is evident in the RD-only versus MD-only comparisons of Math Fluency and Applied Problems (see Figure 2B). It is paralleled in the Co-occurring group as evidenced by the post-hoc contrasts conducted for Research Question 2. This suggests that the difficulties MD children experience with basic and applied math skills become more pronounced as they move into the later elementary grades. This is likely because math is a cumulative subject.

Unlike reading, in which the nature of the requisite reading skills remains largely the same throughout primary education, math skills build on one another and become increasingly conceptually complex as the curriculum progresses. In practice, this means that children face multiple transition points in their math education where they are tasked with developing new skills, many of which depend heavily on strong mastery of previous skills. The pattern we see in our data shows children without disabilities developing basic and applied math skills at a steady rate that may be indicative of curriculum-driven math skill development. In contrast, the slower rate of math skill growth in MD children suggests that as the math curriculum progresses, these children may not be adequately equipped to progress with it. With this in mind, children with MD may benefit greatly from phased support that increases in frequency and/or intensity during transitional points in the math curriculum—such as when multiplication and division are introduced, or when the focus moves from basic operations to applied concepts.

#### RD Patterns—Varying Specificity Over Time

The impairment profile of RD appears to have a more varied combination of specific and general impairments that manifest in both achievement levels and growth patterns. This was evident in the unexpected difficulties the RD-only children exhibited on Calculation, Math Fluency, and Applied Problems. It is not surprising that RD-only children would struggle with applied problems-solving. It is a higher-order math skill that relies heavily on comprehension. Although it is delivered orally, the Applied Problems measure primarily features verbal representations of number words, which are known to draw on language processing (Dehaene, 1992). It is possible that low performance on Applied Problems in RD-only children is a byproduct of reading difficulty. This possibility is supported by the dual finding that RD-only children have similarly steep growth in both Applied Problems (see Figure 2B) and Passage Comprehension (see Figure 2C). In Passage Comprehension, it appears that RD-only children start Grade 1 decidedly lower than MD-only children but grow steadily over time to and catch up to MD-only peers by Grade 5. Passage comprehension is a higher-order skill which relies on sufficient mastery of basic reading skills, such as word-level decoding and fluency, for success. Insufficient basic reading skills have ripple effects in comprehension. It is likely that particularly low comprehension scores in children with RD at the beginning of Grade 1 improve at pace as previously weak basic reading skills strengthen. By extension, comprehension-facilitated applied problem-solving improves as well.

The more surprising finding is that RD-only children show impairment on Calculation and Math Fluency. As measured in this study, these skills are focused squarely on numeracy—both measures exclusively use Arabic digit representations of numbers and involve minimal language processing compared to other measures. Difficulty in these skills is likely the product of domain-general cognitive deficits. The RD-only group’s impairment in math fluency may be the result of domain-general deficits that have been implicated in RD, such as processing speed (Moll et al., 2016; Shanahan et al., 2006) or attentional shifting (Ashkenazi et al., 2013). Whatever be the underlying cause, it appears to be more relevant to early math development than it is to later development. Growth patterns in the RD-only group suggest that poor math fluency at the beginning of Grade 1 resolves over time, reaching levels similar to non-disabled peers around Grade 5.

By contrast, the calculation impairments evident in the RD-only group do not resolve by Grade 5 (see Figure 1D). The RD-only group exhibits calculation difficulty on par with the MD-only group throughout our study period. There are a couple possible explanations for this. It could be a cross-domain manifestation of general cognitive deficits associated with RD. A large meta-analysis by Peng et al. (2022) found that deficits in phonological processing and language comprehension were the primary contributors to RD, but that executive function and visuospatial impairments were also unique contributors. Executive function and visuospatial processing both have known association with math development (Cragg & Gilmore, 2014; Mix & Cheng, 2012). It is possible that RD-related impairments in these processes could be driving the persistent calculation difficulties exhibited by the RD-only children in our study.

Another possibility is that poor reading skills create a barrier to access in math learning that is more evident in Calculation than the other measured math skills. The Math Fluency and Applied Problems assessments primarily feature fundamental math operations, in the context of speed and applied problem-solving, respectively. The Calculation measure assesses an array of increasingly difficult skills that are heavily reliant on thorough, direct, concerted, sequential math instruction. It is possible that the RD-only group’s difficulty with calculation is less about domain-general cognitive impairments and more about having an extra hurdle to navigate in the instructional environment. It is likely that both are contributors, given how multifactorial learning disabilities tend to be.

### Covariates

Although the effects most central to the goals of our analyses were those of the disability group variables, there are some aspects of our covariate effects that warrant discussion. A review of fixed-effects estimates revealed that White racial identity was associated with higher scores on Word ID, and Hispanic ethnicity was associated with lower scores on five outcomes, including all reading outcomes and Math Fluency. It is important to note here that norm-referenced language assessments tend toward racial, cultural, and social bias. This is particularly evident in assessments that leverage vocabulary and general knowledge assumed to be familiar to children (Alptekin, 2006; Banks, 2006; Schon et al., 2008).

One particularly notable finding was the association between non-English language exposure and Calculation and Math Fluency outcomes. Children with multi-language exposure reliably outscored English-only children on these basic math skills. Since the Language variable is not truly a marker of bilingual or dual-language status, very little can be inferred from our results about the relationship between Language and basic math skills. However, the fact that this relationship was found in basic math skills alone presents an interesting line of questioning. Future research could explore whether the cognitive benefits of dual-language exposure and/or bilingualism extend to the math domain. If so, this would be suggestive of general, rather than domain-specific, protective factors associated with multiple-language exposure. This is consistent with the bilingualism literature that has found associations between bilingualism and cognitive benefits in executive function, task switching, and attentional control (Bialystok, 1999; Carlson & Meltzoff, 2008; Diamond et al., 2005), as well as creativity, flexibility, and problem-solving (Adesope et al., 2010; Leikin, 2013).

### Limitations—Complications of Classification

While our classification process was designed with emphasis on precedent in the literature and aligned with classification processes used in clinical and educational practice, it is important to acknowledge that no system of classification can be entirely accurate. A unique challenge of cross-domain research is that commonly used cut scores vary between math and reading domains. For consistency, we used the same cut score for classification of disability in both domains—classifying a score below the 16th percentile as evidence of disability. However, there is growing recommendation for more conservative classification criteria in the MD literature, and 10th percentile has been recommended as a cut score that balances sensitivity and accuracy when identifying MD (Geary, 2011; Geary et al., 2007; Murphy et al., 2007). To address this, we conducted a secondary analysis with a more conservative MD classification cut score to confirm that the pattern of results remained the same.

Our secondary analyses also aimed to help protect against the more general drawbacks of cut score classification. There are important theoretical and philosophical arguments against using numerical cut scores to classify disability in reading or math—see the article by Branum-Martin et al. (2013) for a discussion of these arguments in the specific context of co-occurring reading and math difficulty. One such argument is that when groups are classified using cut scores, the groups themselves—and by extension the differences between groups revealed through comparative analysis—are merely an artifact of the cut score itself (Branum-Martin et al., 2013). Our secondary analyses served, in part, to help distinguish meaningful group differences from those that may be artifacts of the cut score used. With the more conservative MD cut score, the distribution of group membership was altered. Yet, when the same analyses were performed with these altered groups, the pattern of results remained largely the same, suggesting that the observed group patterns generalize beyond the specific cut score used.

While these secondary analyses provide added support, they do not resolve the concern that there are empirically-verified drawbacks to using categorical approaches in the study of reading, math, and co-occurring learning disabilities—many related to the statistical dangers of dichotomizing continuous data (Branum-Martin et al., 2013). Cut scores impose simplicity that is not reflective of the complexity of human performance. Despite the drawbacks, there are distinct practical and methodological limitations that necessitate clear classification boundaries, not the least of which is that in educational practice, limited time and resources necessitate some form of heuristic for ascertaining who should receive intervention. For the purposes of the present analyses, clear numerical cut scores admittedly remove some nuance and complexity but do so in exchange for interpretability and applicability to educational settings.

Lastly, we want to note that our analyses are designed to illuminate broad group patterns that, while informative, are not intended to capture the nuance and complexity of learning disabilities as they manifest in the experiences of individual children. Group classifications are used in our analyses to illuminate patterns and tendencies, but this should not be interpreted as suggesting that children, or the spectra of difficulties and capabilities they manifest, can truly be simplistically categorized.

## Conclusion

Our study explored how key math and reading skill impairments associated with MD and RD manifest over time. Our findings suggest that the math and reading growth trajectories of children with learning disabilities parallel those of children without disabilities. Similarly, the impairment levels and growth trajectories of children with co-occurring MD and RD match the within-domain patterns of children with isolated disabilities. Lastly, there are disparate levels of specificity in MD versus RD. MD impairments are domain-specific, start subtly, and become more pronounced over time, while RD impairments are more general, impact both domains early on, and become more domain-specific over time as some math difficulties resolve.

Our findings have several implications. First, they suggest that developmental change in foundational reading and math skills follows a predictable pattern over time, regardless of skill level. This means that early skill difficulties in children with disabilities do not naturally resolve over time and necessitate targeted intervention. Second, findings suggest that in terms of achievement levels and longitudinal growth, co-occurring MD and RD is functionally the combination of MD-related math difficulties plus RD-related reading difficulties. Third, MD may be especially difficult to catch in early grades because it is characterized by distinctly slow skill growth that takes time to become noticeable. And lastly, in early grades, cases of isolated RD may be difficult to distinguish from co-occurring MD and RD because even in the absence of co-occurrence, RD shows early cross-domain math impairments. Just as cases of isolated MD take a while to fully manifest, cases of isolated RD may take a while to distinguish themselves.

In application, these results are especially important for informing decisions about early grade math intervention. Given these collective findings, it would be beneficial for children who show early signs of math and/or reading difficulty to receive phased math intervention that increases in intensity or frequency when they are introduced to new math concepts. In early grades, this math intervention should provide both linguistic and conceptual support, as our findings suggest that the source of early math impairment (whether math-specific, reading-related, or both) may not be discernible until well into elementary school. As children progress through middle-to-late elementary and their impairment profiles begin to differentiate, intervention approaches can then be adapted to suit.

## Supplemental Material

sj-pdf-1-ldx-10.1177_00222194241312189 – Supplemental material for Specificity, Co-Occurrence, and Growth: Math and Reading Skill Development in Children With Learning Disabilities Supplemental material, sj-pdf-1-ldx-10.1177_00222194241312189 for Specificity, Co-Occurrence, and Growth: Math and Reading Skill Development in Children With Learning Disabilities by Katherine Helene Connors, Emily L. Guertin, Melissa Nichol, Joan M. Bosson-Heenan, Jeffrey R. Gruen and Jan C. Frijters in Journal of Learning Disabilities

sj-pdf-2-ldx-10.1177_00222194241312189 – Supplemental material for Specificity, Co-Occurrence, and Growth: Math and Reading Skill Development in Children With Learning Disabilities Supplemental material, sj-pdf-2-ldx-10.1177_00222194241312189 for Specificity, Co-Occurrence, and Growth: Math and Reading Skill Development in Children With Learning Disabilities by Katherine Helene Connors, Emily L. Guertin, Melissa Nichol, Joan M. Bosson-Heenan, Jeffrey R. Gruen and Jan C. Frijters in Journal of Learning Disabilities

sj-pdf-3-ldx-10.1177_00222194241312189 – Supplemental material for Specificity, Co-Occurrence, and Growth: Math and Reading Skill Development in Children With Learning Disabilities Supplemental material, sj-pdf-3-ldx-10.1177_00222194241312189 for Specificity, Co-Occurrence, and Growth: Math and Reading Skill Development in Children With Learning Disabilities by Katherine Helene Connors, Emily L. Guertin, Melissa Nichol, Joan M. Bosson-Heenan, Jeffrey R. Gruen and Jan C. Frijters in Journal of Learning Disabilities
