# Supplementary material for: Specificity, Co-Occurrence, and Growth: Math and Reading Skill Development in Children With Learning Disabilities
Source: J Learn Disabil. 2025 Feb 9;58(6):411–30. doi: 10.1177/00222194241312189 (PMC12334718; doi:10.1177/00222194241312189)
Supplement: sj-pdf-2-ldx-10.1177_00222194241312189 – Supplemental material for Specificity, Co-Occurrence, and Growth: Math and Reading Skill Development in Children With Learning Disabilities [file sj-pdf-2-ldx-10.1177_00222194241312189.pdf]

JOURNAL OF LEARNING DISABILITIES SUPPLEMENTAL FILE

FIGURE S2. Co-occurring Versus Single Disability Group Differences in Model Predicted Growth on All Outcomes

ARTICLE TITLE: Specificity, Co-occurrence, and Growth: Math and Reading Skill Development in Children with Learning Disabilities

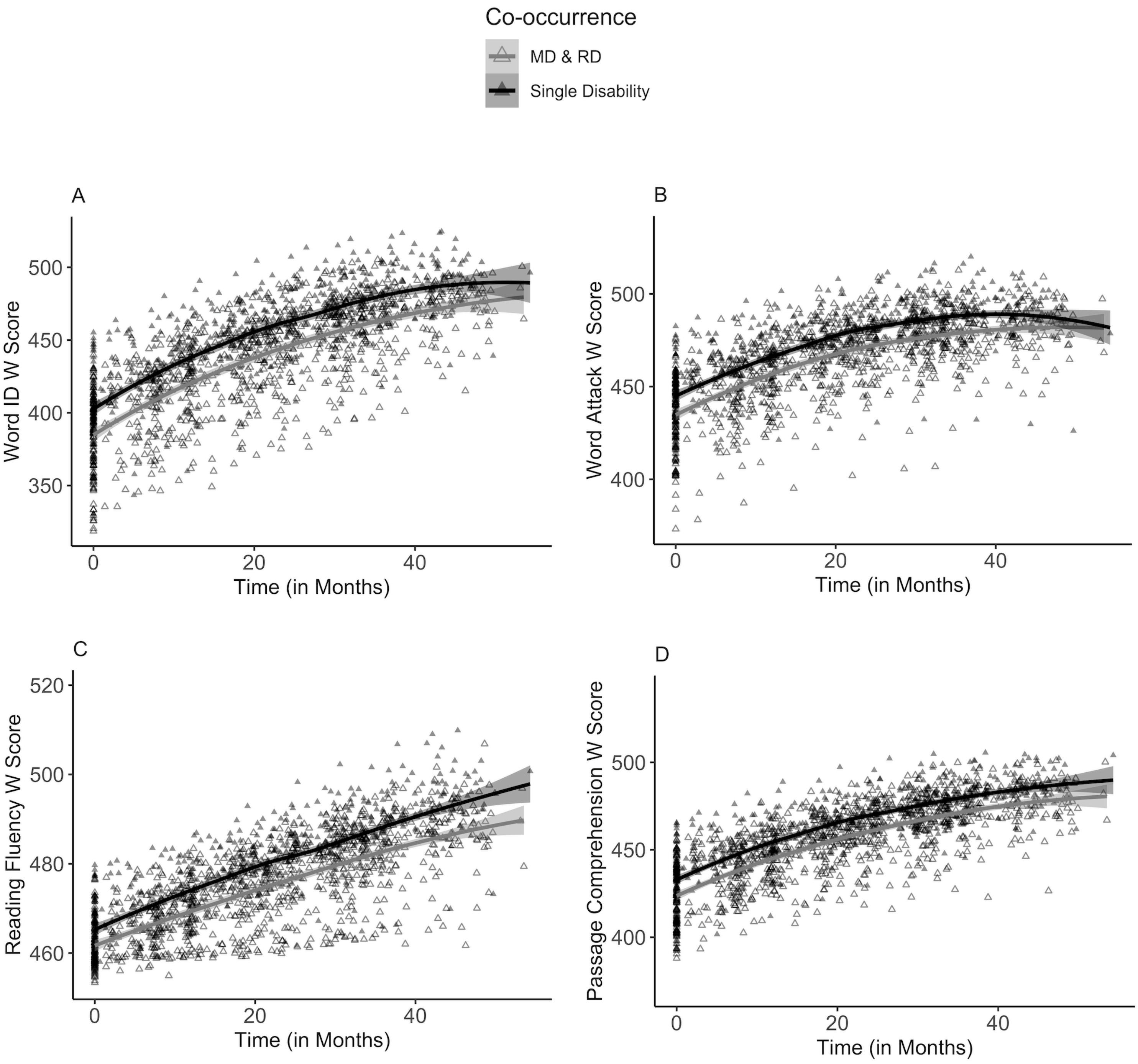

JOURNAL OF LEARNING DISABILITIES SUPPLEMENTAL FILE

FIGURE S2. Co-occurring Versus Single Disability Group Differences in Model Predicted Growth on All Outcomes

ARTICLE TITLE: Specificity, Co-occurrence, and Growth: Math and Reading Skill Development in Children with Learning Disabilities

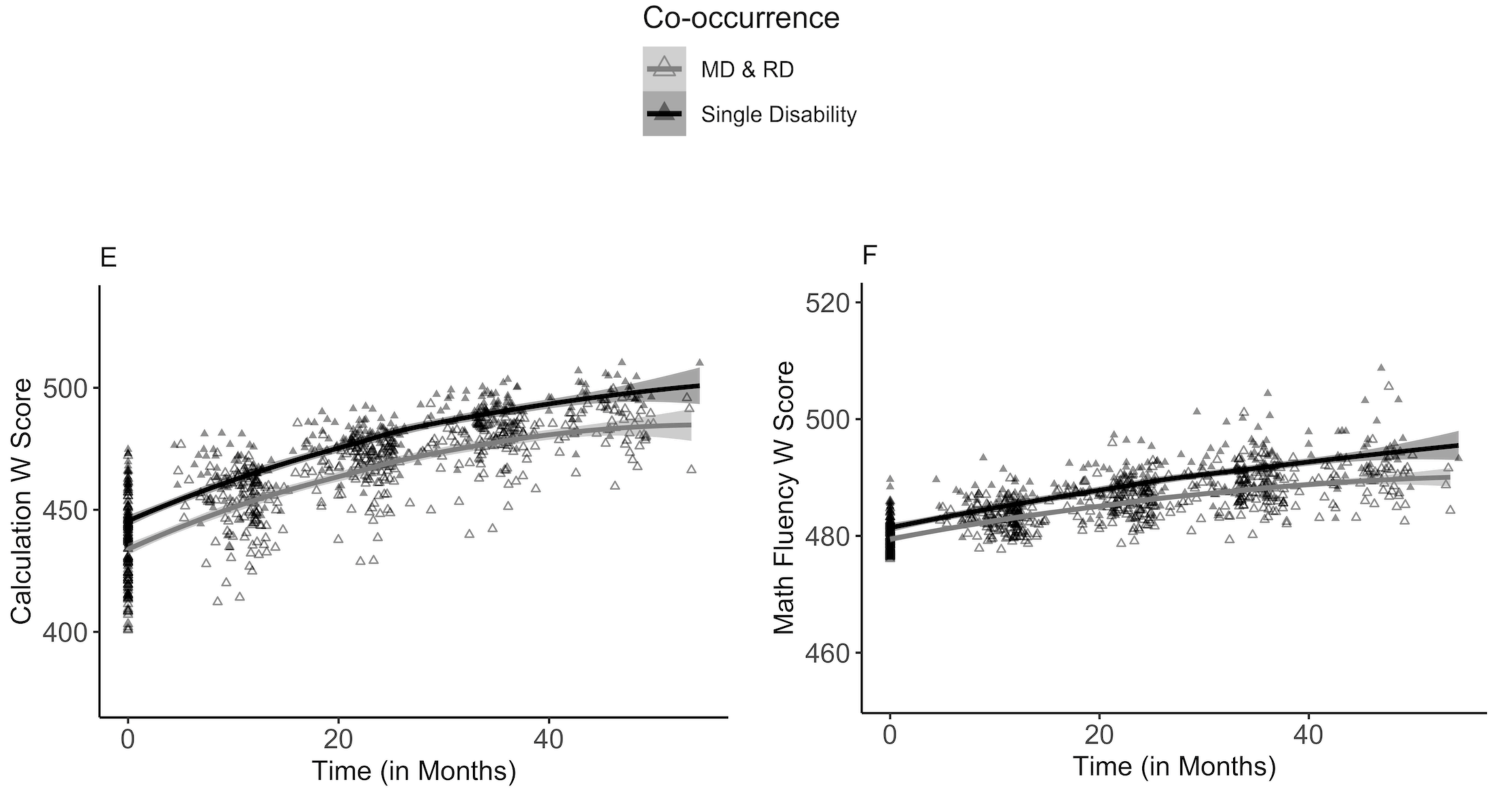

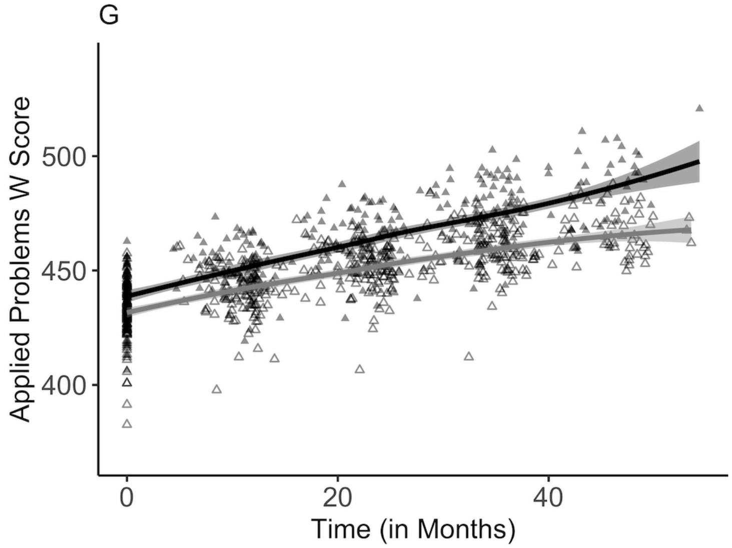

FIGURE CAPTION: Co-occurring versus single disability group growth trajectories on all seven outcome variables plotted by groups with and without disability. Plots show model predicted W scores plotted over time (in months), visualized in R using ggplot2 with loess smoothing for regression lines. Shaded areas represent 95% confidence intervals.
